# Therapeutic Effects of Gelseminum

**Published:** 1870-12

**Authors:** 


THERAPEUTIC EFFECTS OF GELSEMINUM.—Dr. E. A. Anderson, of North Carolina, experimented very extensively during the war with the Gelseminum, in his efforts to find some agent that would serve the purpose of quinine in intermittent and remittent fevers. He now uses this agent — the Gelseminum — almost exclusively in intermittents, especially in children, to whom it is especially adapted, from its absence of any of the unpleasant taste of quinine; he has seldom been disappointed in its effects. He employs the tincture (℥iv. bruised root to Oi. alcohol) or the fluid extract of Tilden. Dose in the proportion of twenty drops to an adult.

It should, like quinine, be given before the expected paroxysm—four to six doses should be given before its onset is expected.

He says, "I have found it a reliable agent in intermittent, remittent and typhoid fevers; acute and chronic rheumatism; in inflammations of the lungs, pleura, and pneumonia. I have, for several years, used it almost exclusively in pneumonia, in place of veratrum, and consider it the best agent in this disease. You can reduce the force and frequency of the circulation with it, as certainly as by venesection, digitalis, or veratrum, without the distressing effects of these agents, while, at the same time, it allays the morbid heat, restlessness, and irritating cough."—*Journal Materia Medica.*

